# Cognitive Function before and during Treatment with Selective Serotonin Reuptake Inhibitors in Patients with Depression or Obsessive-Compulsive Disorder

**DOI:** 10.1155/2016/5480391

**Published:** 2016-08-15

**Authors:** Mehdi Sayyah, Kaveh Eslami, Shabnam AlaiShehni, Leila Kouti

**Affiliations:** ^1^Education Development Center, Ahvaz Jundishapur University of Medical Sciences, Ahvaz, Iran; ^2^School of Pharmacy, Ahvaz Jundishapur University of Medical Sciences, Ahvaz 61357-15794, Iran; ^3^Student Research Committee, Ahvaz Jundishapur University of Medical Sciences, Ahvaz, Iran

## Abstract

*Objectives*. Identification of adverse effects of selective serotonin reuptake inhibitors (SSRIs) is of great importance due to their extensive use in medicine. Some studies have reported the effects of SSRIs on cognitive functions, but the results are conflicting. This study was designed to assess the effect of these drugs on cognition of patients with depression or obsessive-compulsive disorder (OCD).* Methods*. Patients with depression or OCD, naïve to therapy, and candidates of receiving one drug from SSRI class, voluntarily, entered this study. Mini-Mental State Examination (MMSE) test was the tool to assess their cognitive functions. MMSE scores of each patient were recorded prior to taking SSRIs and at weeks 3, 5, and 8 of drug therapy.* Results*. 50 patients met our inclusion criteria, with a baseline mean MMSE score of 23.94. At 3, 5, and 8 weeks of treatment, the mean scores were 22.1, 21.4, and 20.66, respectively. With a *p* value of <0.0001, the gradual decline was statistically significant.* Conclusion*. The MMSE scores of our patients showed a gradual decline over the consecutive weeks after taking SSRI drugs. It seems that the use of SSRIs in patients with depression or OCD, can cause cognitive dysfunction in the acute phase of treatment.

## 1. Introduction

Selective serotonin reuptake inhibitors (SSRIs) are the first-line drug treatment of depression and obsessive-compulsive disorder (OCD). Monotherapy with this class is often recommended for patients primarily diagnosed with these conditions [1, 2].

Despite the potential usefulness of SSRIs, they could cause unwanted side effects such as headaches, weight gain, sexual dysfunction, changes in sleep patterns, and gastrointestinal problems [3]. Originally, SSRIs did not appear to cause cognitive impairment, but nowadays there are conflicting evidences regarding this side effect and many patients complain of memory loss during their course of therapy with SSRIs [4, 5].

The aim of this study is to assess the effect of SSRI drugs on cognitive function of patients with depression or OCD, in acute phase of therapy. In this phase, knowledge and management of side effects are very critical for the patient's compliance to therapy, since these patients might not be motivated enough to cope with adverse effects along with their psychiatric symptoms [6]. In many health problems, drug induced memory loss can be an important cause of medication nonadherence [7, 8].

Current study evaluates the patients' cognitive function, prior to taking SSRIs and throughout the acute course of their therapy.

## 2. Methods

This analytic-descriptive study was conducted in a psychiatric outpatient clinic from April to September 2015. It was permitted by Ahvaz Jundishapur University of Medical Sciences ethics committee. Among patients diagnosed with depression or OCD by a board certified psychiatrist, those who currently were not under any treatment for mental or psychiatric disorders and were candidates of receiving a drug from SSRI class as monotherapy were asked to participate in this study. Inclusion criteria consisted of age between 18 and 60 years, no diagnosed cognitive impairment or mental retardation, no other comorbid disorders in Axis I, the ability to write and read, no visual or auditory impairment, no history of loss of consciousness after head trauma or brain surgery, and no history of using psychoactive drugs such as antidepressants, neuroleptics, benzodiazepines, antiepileptics, or opioids within 14 days prior to the study. Individuals with memory loss complaints in daily functioning would not be included either. If the patient developed any condition in which the use of SSRIs was contraindicated or any disability which interfered with performance of cognitive tests, he/she would be excluded from the study. In addition, patients who stopped their medication for seven consecutive days or did not adhere well with their drug therapy would be excluded. All patients signed a consent form prior to the study.

Several approaches have been developed to help with the diagnosis of cognitive impairment. Some of these tests are Montreal Cognitive Assessment, DemTect, Short Test of Mental Status, Clinical Dementia Rating, and Mini-Mental State Examination (MMSE) [9–12]. Among these, MMSE test is the only valid and reliable test in Farsi; thus, we used this tool for our patients [13, 14]. Aspects of cognitive function assessed by this test include orientation, registration, attention, calculation, recent memory, and language skills. Seyedian et al. translated the Farsi version of MMSE. Its reliability based on Cronbach's Alpha is 81% and a cut-off point of 22 showed a sensitivity of 90% and specificity of 93.5% [13, 14].

The patients' demographic data (age, gender, level of education) were collected in the first visit (week 0 of therapy) and MMSE (a 30-point test) was performed at weeks 0, 3, 5, and 8 of drug therapy. Each patient's drug regimen (type of drug and dosage) remained fixed throughout this 8-week period. If for any reason the patient needed a dose reduction or increase or medication discontinuation, he/she would be excluded from the study. The response to therapy and clinical improvement of OCD/depression signs and symptoms would also be evaluated and recorded.

### 2.1. Statistical Analysis

The data were analyzed using repeated measures one-way ANOVA. Results with a *p* value of less than 0.05 were considered significant. Correlations of MMSE scores and age, sex, and level of education were analyzed by Pearson correlation test, one-way ANOVA test, and paired sample *t*-test, respectively.

## 3. Results

Fifty patients (28 women and 22 men, ages ranging from 20 to 60 years) participated in this study (50% diagnosed with depression and 50% with OCD). They all completed the study and we had no dropout patients. All of them showed signs of response to treatment of their psychiatric disorder at week 8. In order to analyze the correlation of level of education with MMSE scores, we grouped the patients into six different groups. Nine patients were in the elementary group (under 6 years of education), seven were in the middle school group (7–11 years), and 16 patients had a high school diploma. The number of patients with a bachelor, masters, or Ph.D. degree was 11, 6, and 1, respectively. The prescribed drugs for patients were paroxetine (52%), Zoloft (28%), fluoxetine (12%), and citalopram (8%).

Table 1 shows the results of MMSE scores prior to treatment and at weeks 3, 5, and 8. With a *p* value of less than 0.0001, the patients' MMSE scores lowered significantly from their baseline.

Correlation of age and MMSE scores was also assessed and the results showed no significant relationship between them, neither prior to therapy (*p* value = 0.4) nor during it (*p* value = 0.12). MMSE scores among male and female patients had a similar decline trend, when week 8 of treatment was compared with the baseline (95% confidence interval of the difference). Level of education showed no significant correlation with decline of cognitive function (*p* value = 0.14).

## 4. Discussion 

Cognitive dysfunction subsequent to SSRI therapy remains controversial and different studies have reported inconsistent results [4]. Memory loss as a side effect can be overwhelming for patients and leads to medication nonadherence [7, 8]. We used MMSE test for the evaluation of cognitive function of such patients. It is widely used and easy to perform and takes only few minutes of patient's time but it has its limitations as well. Some of these psychometric limitations of MMSE test include ceiling effect, floor effect, and poor distinguishing between mild cognitive impairment and Alzheimer's disease [15]. This test might not be the preferred tool for cognitive assessment of our patients but so far it is the only test in Farsi language that has approved validity and reliability [13, 14, 16, 17].

Due to these limitations, we evaluated the correlation of age and level of education with MMSE scores to clarify the possible influence of these factors on the subjects' cognitive function. Also, since the cut-off points of MMSE might not accurately correlate to the patient's cognitive state, we compared the mean scores before and after therapy and did not group the patients according to the cut-offs [15]. Future studies with the help of other cognitive assessment tools can help to have a more accurate identification of this problem.

Our findings show that patients taking SSRIs experienced statistically significant memory loss during 8 weeks of treatment; age and gender did not influence this result. In contrast, Levkovitz et al. and Culang-Reinlieb et al. reported that some SSRIs have improved memory function in patients with depression [17, 18]. Herrera-Guzmán and colleagues have investigated the effects of SSRIs on cognitive impairment due to depression, in a series of studies. They performed several cognition tests and evaluated the patients in remission and recovery phase. They reported a positive effect of drug therapy with SSRIs in the long term [19]. Deuschle and colleagues performed California Verbal Learning Test on 24 patients with depression and found a low rate of declarative memory loss after 35 days of treatment with SSRIs (the majority of patients had no change from baseline) but no cognitive impairment at long-term therapy (over 12 months). They concluded that the low-grade impairment is due to depressive symptoms and not a side effect [20]. Nevertheless, some evidences strongly supported our results. The study by Popovic et al. showed that over 20% of patients with depression or anxiety disorders reported memory loss after 6 months of SSRI therapy. This was considered a side effect rather than a symptom of untreated depression/anxiety [21]. Wadsworth et al. and Biringer et al. also reported the probability of memory deficit associated with SSRI use [4, 22]. Two other case reports with memory loss subsequent to fluoxetine use have been published in previous years. In one case report, memory loss was reversed when fluoxetine was changed to another SSRI [23, 24]. Although memory impairments can be due to depression itself, memory loss appears to be more likely due to SSRI therapy rather than depression symptoms. Serretti et al. showed that using SSRIs even in healthy individuals leads to cognitive impairment [25]. The memory loss caused by SSRIs has not yet been convincingly explained; however, serotonin appears to play an important role in learning and memory [26]. Serotonin transporter (SERT) expression has been found to be vital for memory development. Decreased expression of this transporter was found in Alzheimer's disease and other memory impairments and dementia [27]. Benmansour et al. showed a decrease in SERT expression within 21 days (3 weeks) following SSRI use [28]. In order to make a better understanding of this issue, Herzallah et al. studied two groups of patients with major depression (one group naïve to medication (16 patients) and one group under successful treatment with paroxetine (15 patients)) and a control group of 25 healthy volunteers, aged 18–60 years. They performed a task evaluating two different aspects of cognition. The results showed that major depression and SSRIs have different positive and negative effects on cognition. SSRIs may have a positive role in striatal function but can deteriorate hippocampus function, needed for generalization. On the other hand, patients with major depression were normal in the latter function, but slow in learning [29].

Although these findings may partly explain the cause of memory loss due to SSRIs, more studies are needed to discover the exact mechanism responsible for this outcome.

We did not evaluate the effect of disease on cognitive function and only targeted the influence of SSRIs on memory. Although all of the patients had higher baseline MMSE scores compared to short-term treatment with SSRIs, their mean score even prior to therapy was lower than normal population (MMSE = 24–26) [30].

## 5. Conclusions

Our data show that in patients with OCD or depression and those who are naïve to SSRI therapy, a gradual decline in their memory function can develop within the first 8 weeks of initiation of drug treatment with SSRIs. This decline did not have a relationship with the patient's age or gender, nor his/her level of education. We suggest further follow-up studies of these patients to identify the persistence of memory loss. Our results were obtained via MMSE test and regardless of the specific drug or disorder. We hope that soon other cognitive function tests would be valid and reliable for Iranian population.

## Figures and Tables

**Table 1:** Results of patients' cognitive assessment prior to and during treatment with SSRs (number of patients = 50).

Weeks of treatment	Week 0	Week 3	Week 5	Week 8
Mean MMSE score	23.94	22.1	21.4	20.66

*p* values of comparing MMSE scores of weeks 3, 5, and 8 with baseline score	—	<0.0001	<0.0001	<0.0001
*p* values of comparing MMSE scores of weeks 5 and 8 with the results of week 3	—	—	<0.0001	0.0709
*p* values of comparing MMSE scores of week 8 with the results of week 5	—	—	—	<0.0001

The trend of decline shows faster decline by 5 weeks and a more subtle decline until week 8.
